# Evaluation of an Automated Wall-mounted Far Ultraviolet-C Light Technology for Continuous or Intermittent Decontamination of *Candida auris* on Surfaces

**DOI:** 10.20411/pai.v9i1.683

**Published:** 2024-05-17

**Authors:** Samir Memic, Claire E. Kaple, Jennifer L. Cadnum, Curtis J. Donskey

**Affiliations:** 1 Department of Systems Biology, Case Western Reserve University School of Medicine, Cleveland, Ohio; 2 Research Service, Louis Stokes Cleveland VA Medical Center, Cleveland, Ohio; 3 Department of Molecular Biology and Microbiology, Case Western Reserve University School of Medicine, Cleveland, Ohio; 4 Department of Medicine, Case Western Reserve University School of Medicine, Cleveland, Ohio; 5 Geriatric Research, Education, and Clinical Center, Louis Stokes Cleveland VA Medical Center, Cleveland, Ohio

**Keywords:** Far ultraviolet-C, environment, *Candida auris*, portable equipment

## Abstract

**Background:**

Technologies that provides safe and eﬀective decontamination of surfaces and equipment between episodes of manual cleaning could be an important advance in eﬀorts to prevent transmission of the emerging fungal pathogen *Candida auris*.

**Methods:**

We tested the efficacy of a novel wall-mounted far ultraviolet-C (UV-C) light technology that delivers far UV-C, when people are not detected within the field of illumination, against *C. auris* isolates from clades I, II, III, and IV using a quantitative disk carrier test method. In an equipment room, we examined the efficacy of the technology in reducing an isolate of *C. auris* from clade IV inoculated on multiple sites on portable devices.

**Results:**

The far UV-C technology reduced isolates from all 4 clades of *C. auris* by >3 log_10_ colony-forming units (CFU) aﬅer an 8-hour exposure on steel disks. For the clade IV isolate, similar reductions were achieved on glass and plastic carriers. In the equipment room, the technology reduced *C. auris* inoculated on multiple sites on portable equipment by >2 log_10_ CFU in 4 hours.

**Conclusions:**

The far UV-C technology could be useful for decontamination of surfaces and equipment between episodes of manual cleaning. Additional studies are needed to evaluate the use of the technology in clinical settings.

## INTRODUCTION

*Candida auris* is a globally emerging multidrug-resistant fungal pathogen that is classified as an urgent threat by the Centers for Disease Control and Prevention [1]. In the United States, *C. auris* cases have increased steadily in recent years with documented spread to 28 states and Washington DC by the end of 2022 [2]. Contaminated surfaces and reusable medical equipment have been implicated as important sources of transmission [3–6]. Therefore, thorough cleaning and disinfection of surfaces in patients' rooms at least daily and of shared equipment aﬅer each use is recommended [1]. However, adequate cleaning and disinfection is challenging because personal items and patient care equipment are oﬅen present in occupied patient rooms, and surfaces near colonized patients rapidly become re-contaminated aﬅer disinfection (ie, within 4 hours) [7].

A technology that provides safe and eﬀective decontamination of surfaces and equipment between episodes of manual cleaning could be an important advance in eﬀorts to prevent transmission of *C. auris*. One promising candidate technology is far ultraviolet-C (UV-C) light (200 to 230 nm) [8]. Far UV-C light is strongly absorbed by proteins and other biomolecules, resulting in minimal penetration into skin or eye tissues [8]. There is growing evidence from animal model and human volunteer studies that far UV-C doses within threshold limit values proposed by the American Conference of Governmental Industrial Hygienists and the International Commission on Non-Ionizing Radiation Protection may be safe [8–13]. We previously demonstrated that far UV-C was eﬀective against *C. auris* isolates from clades II and III; although, the reductions were relatively modest in comparison to vegetative bacterial pathogens (ie, 0.5 to 2.9 log_10_ colony-forming unit reductions in 45 minutes versus >3 log_10_ CFU reductions for vegetative bacteria) [14]. Here, we conducted a more comprehensive evaluation of the efficacy of a far UV-C technology against strains from all 4 clades of *C. auris*. The technology tested is novel in that it detects people and can be programmed to stop or reduce far UV-C delivery when people are present within the area of exposure.

## METHODS

### Description of the Far UV-C Light Technology

**Figure 1.**
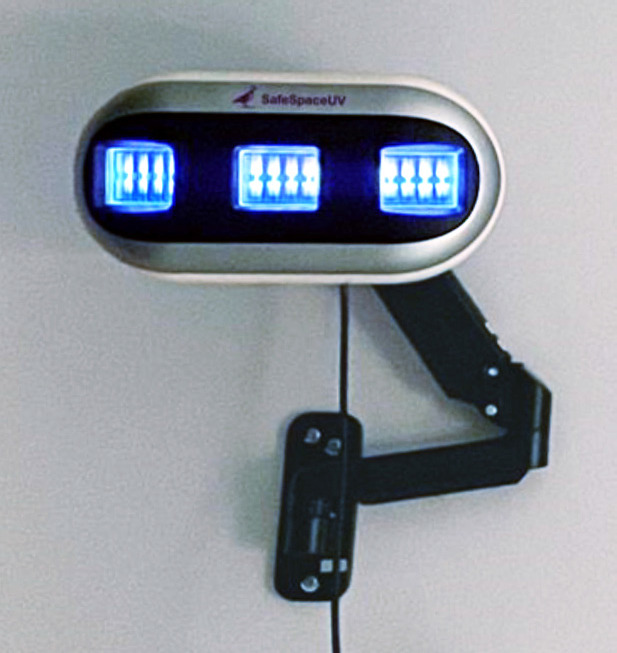
Picture of the wall-mounted far ultraviolet-C device.

The far UV-C technology (Myna Life Technologies, Inc.) uses 3 krypton-chloride excimer lamps that emit a primary wavelength of 222 nm with filters to block emitted wavelengths >230 nm [14]. Figure 1 shows a picture of the device. Each device contains 3 lamps with a field of illumination of 60° per lamp [14]. Two wall-mounted devices are recommended for a typical patient room or equipment room. For this study, the devices were mounted on posts. The device includes proprietary sensors that detect people within the field of illumination, including individuals remaining motionless. For this study, the device was programmed to automatically discontinue all far UV-C light delivery when people enter the field of illumination, to stay oﬀ while people are present, and to resume output 30 seconds aﬅer people exit the field of illumination. The study protocol was approved by the Research and Development and Biosafety Committees at the Louis Stokes Cleveland VA Medical Center (protocol number 1584025).

### *Candida* Species Test Organisms

The *C. auris* test strains included isolates from 4 phylogenetic clades, including AR-0381 (Clade II; East Asia origin), AR-0389 (Clade I; South Asia origin), AR-0383 (Clade III; Africa origin), and AR-0385 (Clade IV; South America origin). Isolates from 4 clades were studied because there are potential diﬀerences in susceptibility to UV-C light. In previous studies, *C. auris* isolates from clades III and IV (AR-0383 and AR-0385) formed aggregates and exhibited reduced susceptibility to 254 nm UV-C light in comparison to a clade II isolate (AR-0381) [15, 16].

For comparison, we tested *Candida albicans* American Type Culture Collection (ATCC) strain 10231, *Candida glabrata* clinical strain MRL#9547, and a clinical *Candida parapsilosis* strain from the Cleveland VA Medical Center.

### Efficacy of Far UV-C Light in Reducing the *Candida* Species Test Strains on Carriers

Testing was conducted in a 25.6 m^3^ room. Two devices were positioned at opposite corners of one wall, 2 m from the floor, angled toward the center of the room, with 1.6 m of space between the devices. With all 6 lamps from the 2 devices operating continuously, the calculated doses of far UV-C delivered over 8 hours at 2 and 3 m from the lamps were 230 and 150 mJ/cm^2^, respectively [14].

We tested the efficacy of the technology against the test organisms using a modification of the American Society for Testing and Materials (ASTM) standard quantitative disk carrier test method (ASTM E 2197–02) and standard practice for determining antimicrobial efficacy of ultraviolet germicidal irradiation against microorganisms on carriers with simulated soil [17, 18]. A soil load of 5% fetal calf serum was used. A 10 µL inoculum containing ~4 log_10_ CFU of the test organism was spread to cover 20 mm steel disks. The disks were oriented horizontally 0.8 m high. All experiments were completed in triplicate. Disks were processed as previously described [17]. Log_10_ reductions were calculated in comparison to untreated controls. For testing on carriers, a 3 log_10_ or greater reduction in the test organisms in comparison to untreated controls was considered eﬀective [14].

An initial set of experiments was conducted with the *C. auris* clade I isolate to assess eﬀectiveness over time at diﬀerent distances from the devices. The inoculated disks were placed 1, 2, or 3 m from the devices. Exposure times of 0.75, 4, 8, 24, and 72 hours were tested. A radiometer (UIT2400 Handheld Light Meter for 222 nm, Ushio America) was used to measure irradiance at 1, 2, and 3 m from the devices. Based on the initial results, all the remaining organisms were tested at 2 m from the devices with exposure times of 4, 24, and 72 hours.

### Inactivation Rate Constants of Far UV-C for the Test Isolates

We determined the inactivation rate constants for the test isolates using a modification of the methods of Lemons et al [19] and Vitzilaiou et al [20]. A 5 mL suspension containing ~10^6^ *andida* species CFU per mL water was added to a petri dish with a magnetic stirrer set at 200 rpm. The suspension was exposed to increasing doses of far UV-C delivered by a single far UV-C device. The doses were 0, 25, 50, 75, 100, 125, 150, and 175 mJ/cm^2^. Following exposure, aliquots of the suspension were plated on selective media to quantify *Candida* species as previously described [17]. Experiments were repeated twice with triplicate samples for each test point. A dose-response curve was plotted by graphing the survival fraction at each far UV-C dose. The inactivation rate constants (k-values) were calculated as previously described [19, 20]. The k-value is inversely related to the dose required to obtain a specific survival fraction.

### Efficacy of the Far UV-C Technology in Reducing *C. auris* on Different Types of Surfaces

The clade IV isolate was selected for subsequent testing because the Environmental Protection Agency (EPA) recommends the use of *C. auris* AR #0385 (clade IV) as the test strain for testing the efficacy of disinfectants [21]. To assess the impact of diﬀerent types of surfaces on the efficacy of far UV-C, experiments were conducted with the AR #0385 isolate inoculated onto steel disks, plastic coupons (acrylonitrile butadiene styrene polymer), and glass slides. The carriers were placed 2 m from the devices with exposure times of 0.75 and 4 hours.

### Efficacy of the Far UV-C Technology in Reducing *C. auris* on Portable Medical Equipment

For these experiments, testing was conducted in a 35.2 m^3^ equipment storage room. Two devices were placed on opposite sides of the room at a height of 2 m. The devices were placed on opposite sides of the room to reduce the potential for shaded areas to receive suboptimal far UV-C dosing. A workstation-on-wheels, portable vital signs unit, and wheelchair were inoculated on 2.5 cm diameter circular areas with 10 µL containing 10^4^ CFU of *C. auris* AR #0385 in 5% fetal calf serum; 2 to 3 sites were inoculated for each type of equipment including sites on the side that would not be in direct line of sight of the far UV-C light. The test sites ranged from 1.5 to 2.2 m from the nearest device. Aﬅer 0.75 and 4 hours of exposure, the inoculated sites were sampled with pre-moistened cotton-tipped swabs. The swabs were processed to quantify *C. auris* as previously described [3]. Testing was completed in triplicate. Log_10_ reductions were calculated in comparison to untreated control surfaces. For inoculated equipment, a 2 log_10_ or greater reduction compared with untreated control surfaces was deemed eﬀective as an adjunct to standard cleaning and dis-infection.

### Discontinuation of Far UV-C Output When People are Within the Area of Far UV-C Exposure

To assess the feature that discontinues far UV delivery when people are within the area of far UV-C exposure, research personnel entered the equipment room 10 times while the device was operating, stood with minimal motion at multiple locations in the vicinity of the equipment for 5 minutes, then exited the room. Personnel determined if the device was on or oﬀ based on visual assessment (ie, the lamps emit visible light when operating) and by carrying the handheld radiometer to measure irradiance.

### Data Analysis

A one-factor analysis of variance (ANOVA) was used to test for significant diﬀerences in k-values among the diﬀerent *Candida* species isolates. A one-way ANOVA was used to compare the log_10_ reductions for *C. auris* clade IV on steel, plastic, and glass carriers. Data were analyzed using R version 3.5.0 soﬅware (The R Foundation for Statistical Computing, Vienna, Austria).

## RESULTS

### Reduction in the Recovery of the *Candida* Species Over 72 Hours of Far UV-C Exposure

**Figure 2.**
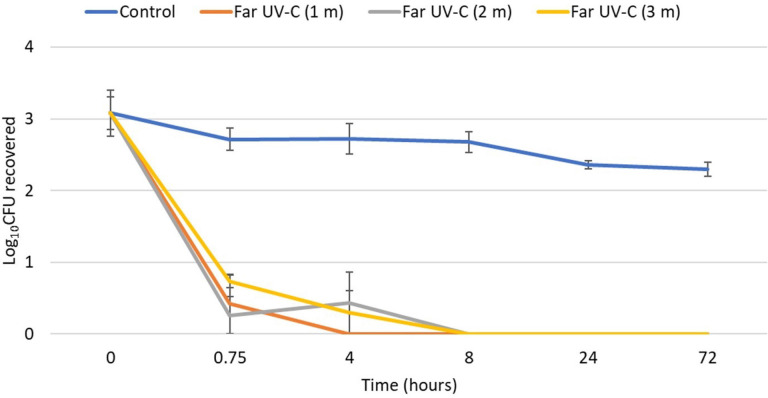
**Efficacy of 2 far ultraviolet-C (UV-C) devices in reducing a clade I isolate of *Candida auris* on steel disk carriers at 1, 2, and 3 m from the devices over 72 hours.** The 2 devices were positioned at opposite corners of one wall 2 m from the floor, angled toward the center of the room, with 1.6 m of space between the devices. Control carriers were unexposed to far UV-C. CFU, colony-forming unit.

As shown in Figure 2, the clade I isolate was reduced by >2 log_10_ CFU aﬅer 0.75 hours of exposure at 1, 2, and 3 m from the far UV-C devices. Aﬅer 8 hours of exposure, no *C. auris* was recovered at 1, 2, or 3 m. Irradiance readings at 1, 2, and 3 m from the devices were 9.0, 9.4, and 5.8 µW/cm^2^. All the remaining test organisms were reduced by >3 log_10_ CFU aﬅer 8 hours and to undetectable levels aﬅer exposure for 24 and 72 hours of exposure at 2 m from the devices.

### Inactivation Rate Constants of Far UV-C for the Test Isolates

**Figure 3.**
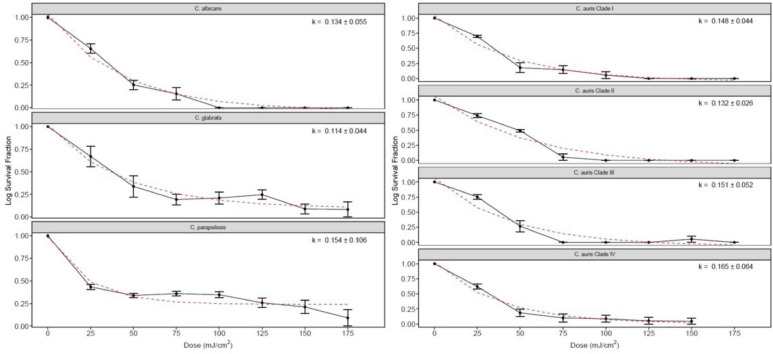
**Far ultraviolet-C (UV-C) dose-response curves and the inactivation rate constants (k-values ±SE) for each of the Candida species isolates.** The survival fraction at each far UV-C dose is represented by black dots. The red dashed lines represent the best-fit line using the exponential decay model. There were no statistically significant diﬀerences in the k-values among the *Candida* species tested (*P*=0.44).

Figure 3 shows the far UV-C dose-response curves and the inactivation rate constants (k-values) for each of the *Candida* species isolates. The survival fraction at each far UV-C dose is represented by black dots, and the red dashed lines represent the best-fit line using the exponential decay model. There were no statistically significant diﬀerences in the k-values among the *Candida* species tested (*P*=0.44; one-factor ANOVA). The k-values for the C. auris isolates ranged from 0.114 to 0.165. There was no substantial reduction in any of the *Candida* species isolates for controls not exposed to far UV-C.

### Impact of Different Types of Surfaces on Efficacy of Far UV-C

**Figure 4.**
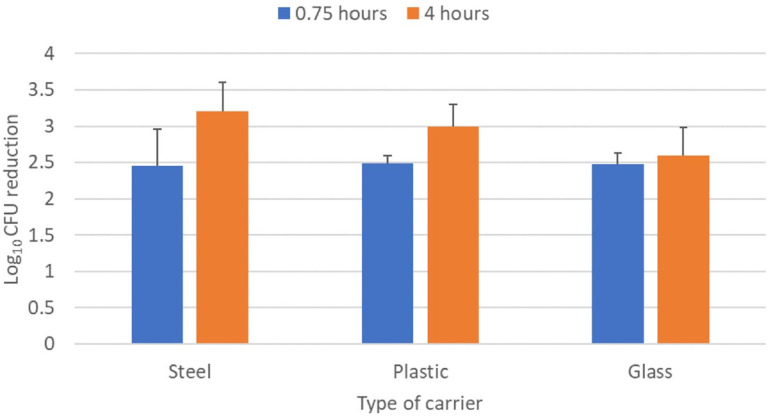
**Efficacy of 2 far ultraviolet-C (UV-C) devices in reducing a clade I isolate of *Candida auris* on steel disk, plastic, and glass carriers.** The carriers were placed 2 m from the devices and exposed to far UV-C for 0.75 or 4 hours. Control carriers were unexposed to far UV-C. CFU, colony-forming unit.

Figure 4 shows a comparison of the log_10_ CFU reductions of the *C. auris* clade IV isolate on steel, plastic, and glass carriers. There were no significant diﬀerences in the reductions achieved with each type of carrier aﬅer 0.75 and 4 hours of far UV-C exposure (*P*>0.05).

### Efficacy in Reducing *C. auris* on Portable Medical Equipment

**Figure 5.**
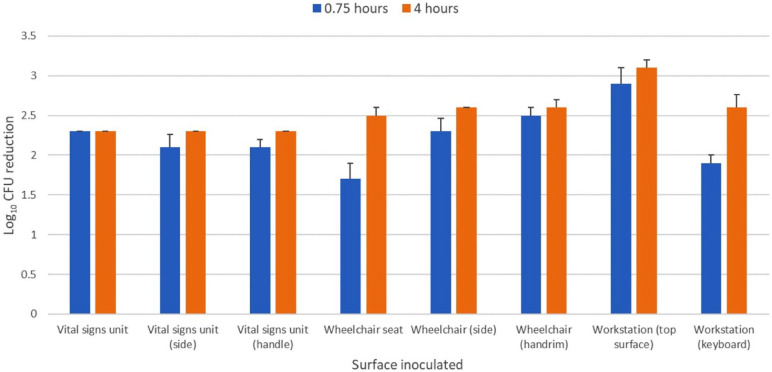
**Efficacy of 2 far ultraviolet-C (UV-C) devices in reducing a clade IV isolate of *Candida auris* inoculated on portable medical devices in an equipment room.** The 2 devices were placed on opposite sides of the room at a height of 2 m. The exposure time was 0.75 and 4 hours and test sites ranged from 1.5 to 2.2 m from the nearest device. Control sites were unexposed to far UV-C. CFU, colony-forming unit.

Figure 5 shows the efficacy of the far UV-C technology in reducing the *C. auris* clade IV isolate on a workstation-on-wheels, portable vital signs unit, and wheelchair in an equipment room. *C. auris* was reduced by >2 log_10_ CFU on all inoculated sites aﬅer 4 hours of exposure.

### Evaluation of the Safety Feature that Discontinues Far UV-C Output When People are Present

The far UV-C devices in the equipment room consistently turned oﬀ when research personnel walked into the areas of far UV-C exposure and remained oﬀ while they stood still at multiple locations in the vicinity of the portable equipment. The devices turned back on 30 seconds aﬅer personnel exited the area of far UV-C delivery. Based on irradiance readings, there was no substantial exposure to far UV-C light (ie, consistent readings of 0 µW/cm^2^) during entries into the room.

## DISCUSSION

We found that an automated wall-mounted far UV-C technology was eﬀective in reducing isolates from the 4 major clades of *C. auris* by >3 log_10_ CFU aﬅer an 8-hour exposure on steel disks and to undetectable levels aﬅer 24 and 72 hours of exposure. The k-values of the *C. auris* isolates for far UV-C ranged from 0.114 to 0.165. These k-values are equivalent to k-values previously reported for the same *C. auris* isolates from the 4 major clades for 254 nm UV-C light (range, 0.130 to 0.176)[19]. For the clade IV isolate, similar reductions were achieved on glass and plastic carriers in 4 hours. In an equipment room, the technology reduced the clade IV *C. auris* isolate on multiple sites inoculated on real-world equipment by >2 log_10_ CFU in 4 hours. These findings suggest that the far UV-C technology could be a useful addition to current approaches to address environmental contamination with *C. auris.*

Safety is an important concern for all UV-C technologies [9]. For the current study, the technology was modified to provide automated delivery of far UV-C only when people were not present. This modification would provide adequate far UV-C doses in areas such as portable equipment rooms that are occupied infrequently or procedure or clinic rooms that may be unoccupied for several minutes between patients. In contrast to standard 254 nm UV-C, if accidental short-term exposure to far UV-C did occur, there would be relatively little risk because such exposure would be below the 8-hour threshold limit values proposed for far UV-C exposure (161 mJ/cm^2^ for eyes and 479 mJ/cm^2^ for skin) [8]. For patient rooms or other frequently occupied areas, the device can be programmed to automatically reduce or discontinue far-UV-C exposure by turning oﬀ one or more light modules as needed based on the proximity of people to keep exposure below a thresh-old limit value of 160 mJ/cm^2^ per 8 hours [8]. Prior to considering routine implementation of far UV-C in occupied areas, additional evaluations of long-term safety are needed. Such evaluations are currently being conducted in clinical settings.

Our study has some limitations. First, we evaluated efficacy on carriers and portable equipment inoculated with *C. auris*. Additional studies are needed in real-world settings where contamination is due to shedding by colonized patients. Second, only 45-minute and 4-hour exposures were tested in the equipment room. Longer exposure times would be anticipated in equipment rooms that are occupied infrequently. Third, it is not known if the reductions in contamination that were achieved will be sufficient to reduce the risk for transmission of *C. auris*. Fourth, although we focused on *C. auris*, patients are oﬅen co-colonized with *C. auris* and other healthcare-associated pathogens [7]. In previous studies, far UV-C light has been shown to be eﬀective against other healthcare-associated pathogens [14]. Finally, we did not compare the efficacy of the far UV-C technology with other potential technologies that might provide continuous decontamination of surfaces [9]. Products such as continuously active quaternary ammonium disinfectants may also be eﬀective in reducing *C. auris*, but may have limitations (eg, easily removed by wiping, efficacy may vary with method of application) [22, 23].

In summary, our findings suggest that the far UV-C technology we studied could be useful for decontamination of surfaces and equipment between episodes of manual cleaning. Given that *C. auris* is classified as an urgent threat, there is an urgent need for additional studies to evaluate the use of the technology in clinical settings.
